# Clathrin-mediated endocytosis is a candidate entry sorting mechanism for *Bombyx mori* cypovirus

**DOI:** 10.1038/s41598-018-25677-1

**Published:** 2018-05-08

**Authors:** Fei Chen, Liyuan Zhu, Yiling Zhang, Dhiraj Kumar, Guangli Cao, Xiaolong Hu, Zi Liang, Sulan Kuang, Renyu Xue, Chengliang Gong

**Affiliations:** 1 School of Biology & Basic Medical Science, Soochow University, Suzhou, 215123 China; 2 National Engineering Laboratory for Modern Silk, Soochow University, Suzhou, 215123 China

## Abstract

*Bombyx mori* cypovirus (BmCPV), a member of the *Reoviridae*, specifically infects silkworms and causes extensive economic losses to the sericulture industry. To date, the entry mechanism of BmCPV into cells is unclear. Here we used electron microscopy to study the route of entry of BmCPV into cells, and the results demonstrated that the entry of BmCPV into BmN cells was mediated by endocytosis. Blocking the entry pathway with four endocytosis inhibitors, including dansylcadaverine, chlorpromazine, genistein, and PP2, significantly decreased the infectivity of BmCPV. This indicates that BmCPV enters BmN cells via endocytosis, and that clathrin-mediated sorting is the predominant entry method. After the relative expression levels of *clathrin heavy chain* (clathrin, GenBank accession No. NM_001142971.1) and the *adaptor protein complex-1 gamma subunit AP*-1 (AP-1, GenBank accession No. JQ824201.1), which are involved in clathrin-mediated endocytosis, were inhibited by RNA interference or abolishing the functions of clathrin and AP-1 with their corresponding antibodies, the infectivity of BmCPV was reduced significantly, which suggests that clathrin-mediated endocytosis contributed to the entry of BmCPV into cells. Our findings suggest that the clathrin-mediated endocytosis pathway is a candidate for the development of therapeutics for silkworm cytoplasmic polyhedrosis.

## Introduction

Cytoplasmic polyhedrosis viruses (CPVs) are the second major group of insect pathogens belonging to the genus *Cypovirus* in the family *Reoviridae*^1^. CPVs are double-stranded RNA viruses with segmented genomes, and the multiplication of CPVs occurs in the cytoplasm of host cells. The genus *Cypovirus*is classified into 19 distinct species (electropherotypes)^2^. *Bombyx mori *cypovirus (BmCPV) is a representative species of cypovirus 1, which multiplies in the epithelial cytoplasm of the silkworm midgut and causes silkworm cytoplasmic polyhedrosis^3^. BmCPV particles have an icosahedral symmetry, with a diameter of approximately 60 to 80 nm, and they lack an outer capsid layer instead of having a single-shelled, turreted structure. The BmCPV genome is composed of 10 discrete equimolar double-stranded RNA segments (S1–S10)^4^ and each segment has a single open reading frame^5^. BmCPV has six viral structural proteins, namely VP1, VP2, VP3, VP4, VP6, and VP7, which are encoded by the S1, S2, S3, S4, S6, and S7 genes, respectively. The major capsid protein is VP1, and it functions to assemble virus particles. The S2 gene encodes RNA-dependent RNA polymerase, which catalyzes the synthesis of viral mRNA. The nonstructural proteins (NSPs) p101 (NSP5), p44 (NSP8), NS5 (NSP9), and polyhedrin are encoded by the S5, S8, S9 and S10 genes, respectively. Among these proteins, VP3, a spike protein, was predicted to bind to a receptor located on the surface of host cells during the CPV infection process^6^. Previous studies predicted functions of the proteins encoded by BmCPV^5^, and the innate immune response of the silkworm to BmCPV infection was clarified, while gene transcript changes in the midgut of BmCPV-infected and normal silkworm larvae were determined by RNA sequencing and microarray analyses^7,8^; however, the pathway of entry of BmCPV into midgut cells remains unknown. Electron microscopy observations found that intact BmCPV virions enter midgut epithelial cells of the silkworm by directly penetrating the cell membrane^9^; however, the cell entry mechanisms of BmCPV are still poorly understood, which has hampered efforts to develop a safe and effective drug against BmCPV.

Mammalian orthoreoviruses (reoviruses) are the prototype members of the *Reoviridae* family of non-enveloped viruses^10^. Cell entry of orthoreoviruses has been well studied. Reoviruses initially bind to carbohydrates located on the cell surface, followed by attachment to their receptor, junction adhesion molecule-A (JAM-A), and then the virus is internalized by clathrin-mediated endocytosis using a process that is dependent on β1 integrin^11,12^. Rice dwarf virus (RDV) is a member of the genus *Phytoreovirus* in the family *Reoviridae*, and the entry of RDV into *Nephotettix cincticeps* cells was demonstrated to depend on clathrin-mediated endocytosis, which is also true for mammalian reoviruses^13^, suggesting that endocytosis plays an important role in the early events of reovirus infection.

Inhibitors of endocytosis have been used to investigate the cell entry of viruses^13,14^. Dansylcadaverine can suppress reovirus internalization by receptor-mediated endocytosis^14^. Chlorpromazine inhibits clathrin-mediated endocytosis by preventing the assembly and disassembly of clathrin lattices on cell surfaces and on endosomes^15^. Genistein is a broad-spectrum tyrosine kinase inhibitor that interferes with caveolae-mediated endocytosis by inhibiting the internalization of viruses into cells, and it has been reported that it can induce apoptosis and autophagy in cancer cells^16,17^. 4-Amino-5-(4-chlorophenyl)-7-(t-butyl) pyrazolo [3, 4-d] pyrimidine (PP2) is a specific Src-family kinase inhibitor^18^. It has been proven that Src kinase can regulate the proper sorting of virus particles in the endocytosis pathway, and that it helps disassemble viruses, which promotes viral cell entry. PP2 does not obstruct virus internalization by impairing viral attachment to the cell surface, but it inhibits early steps of viral entry, leading to anomalous transport of virus particles to lysosomes^19^.

To date, there is no silkworm variety that is highly resistant to BmCPV; thus, protecting silkworms from BmCPV infection is conducted by inactivating BmCPV virions that exist in the rearing environment using disinfectors, and by enhancing the resistance of silkworms through feeding and management during cocoon production; however, the prevention and control of silkworm cytoplasmic polyhedrosis in sericulture remains a large problem.

In the present study, we studied the route of entry of BmCPV into cells. We found that clathrin-mediated endocytosis plays an important role in the entry of BmCPV into cells, and that blocking the entry pathway with endocytic inhibitors (dansylcadaverine, chlorpromazine, genistein, and PP2) reduced BmCPV infectivity *in vitro* and *in vivo*. These results provide a novel basis by which to screen for effective therapeutic medicines that prevent silkworm cytoplasmic polyhedrosis.

## Results

### BmCPV particles are internalized into BmN cells via endocytosis

**Figure 1:**
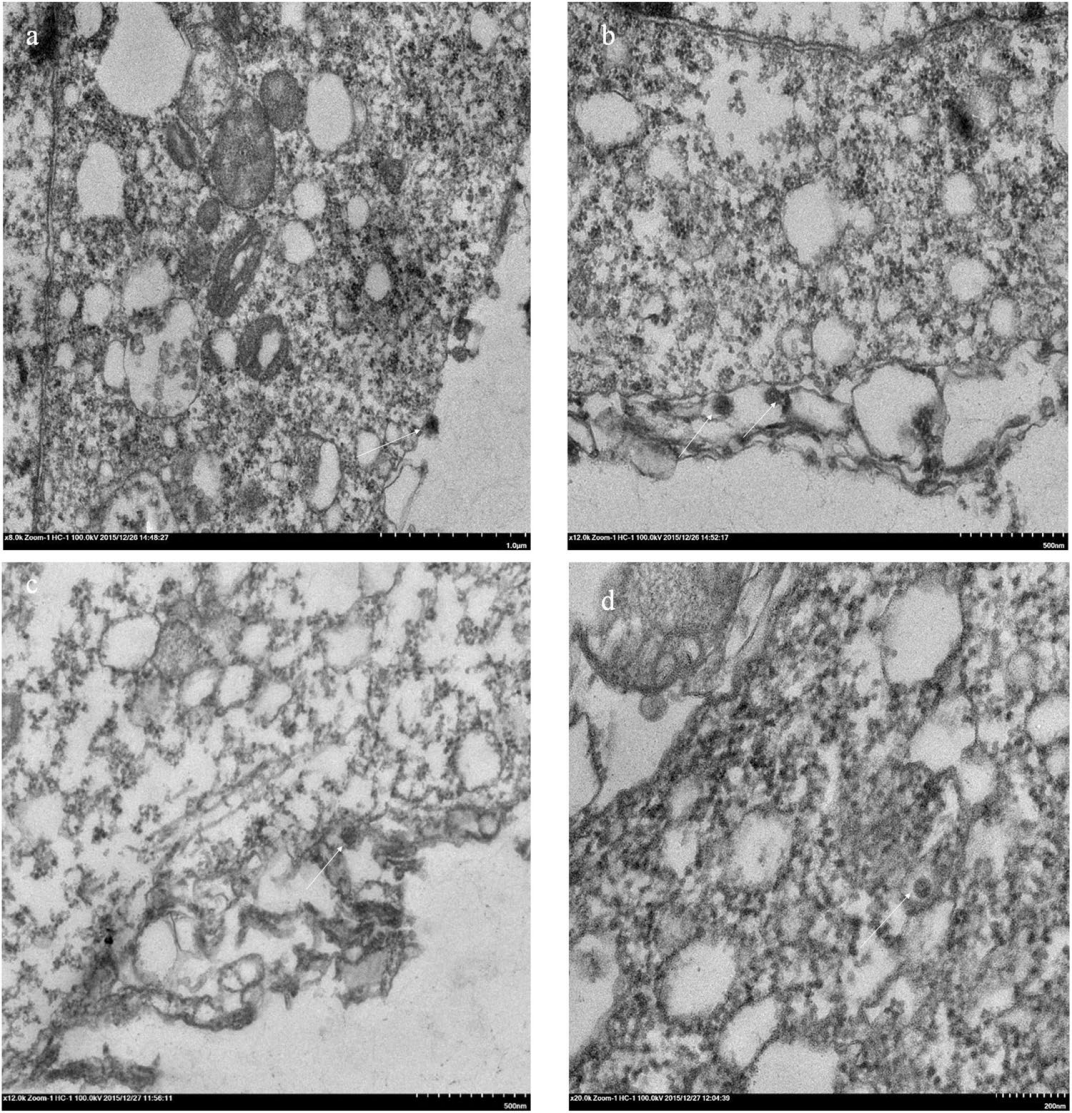
Electron microscopy observation of the early stage of BmCPV infection. (**a** and **b**) Attachment of BmCPV particles to the membranes of BmN cells at 30 min p.i.; (**c**) BmCPV particles were invaginated into the plasma membrane at 1.5 h p.i.; (**d**) BmCPV particles were delivered into an endosome-like structure at 1.5 h p.i.

To determine whether BmCPV particles are internalized into BmN cells via endocytosis, BmN cells were inoculated with BmCPV, and they were investigated by transmission electron microscopy at an early stage of infection. The results showed that BmCPV particles, with a diameter of approximately 60–70 nm, attached to the surfaces of the BmN cells at 30 min post-inoculation (p.i.) (Fig. 1a,b). Subsequently, the plasma membrane invaginated, forming a pocket containing BmCPV particles at 1.5 h p.i. (Fig. 1c), and then the internalized virions were delivered to an endosome-like structure (Fig. 1d), suggesting that the entry of BmCPV into cells might be mediated by endocytosis.

### Endocytic inhibitors reduce the infectivity of BmCPV in BmN cells

**Figure 2:**
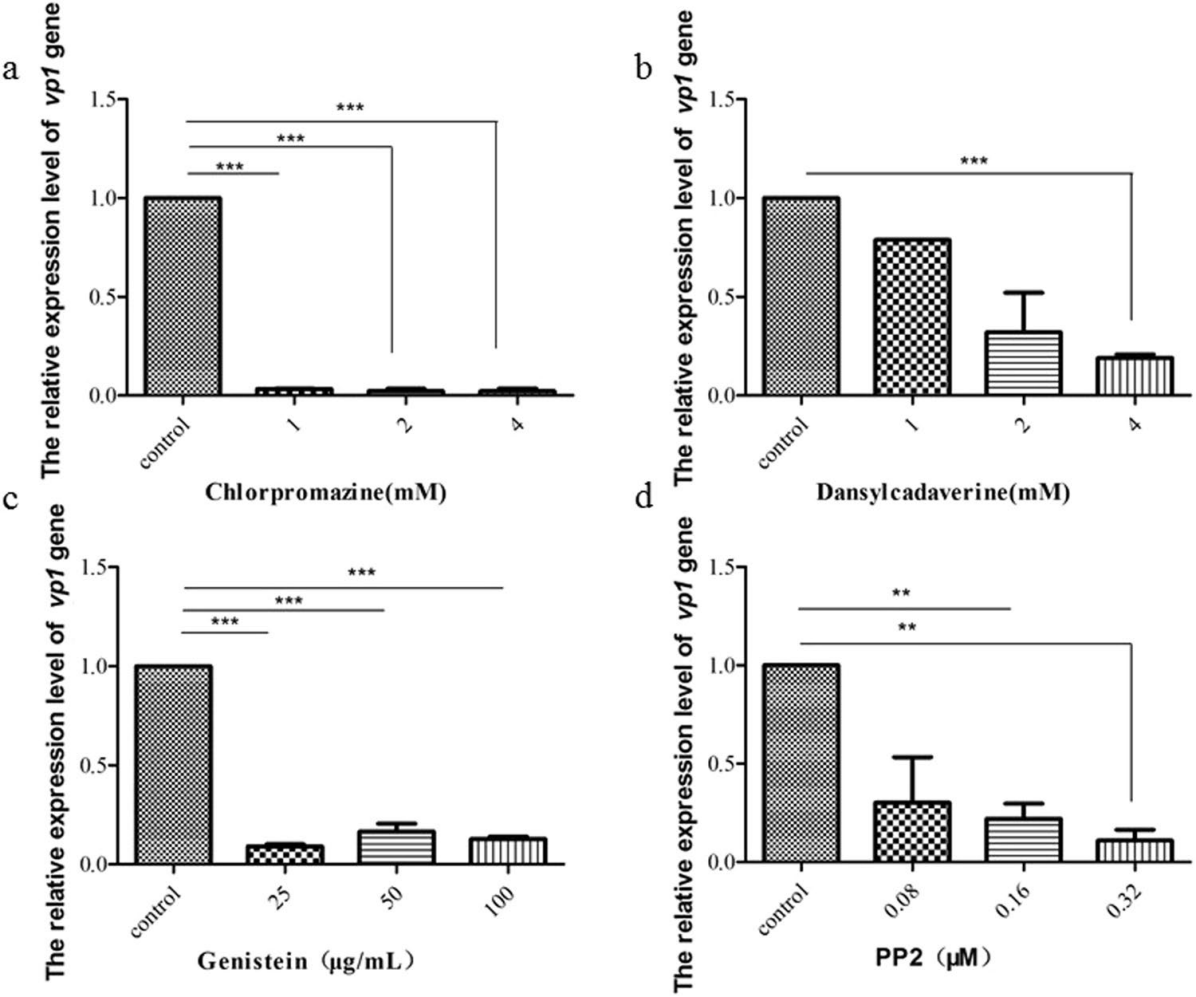
The decrease of the infection of BmCPV in BmN cells with the endocytic inhibitors. BmN cells (2 × 10^5^) were incubated in TC-100 medium containing (**a**) chlorpromazine (1, 2, and 4 mM), (**b**) dansylcadaverine (1, 2, and 4 mM), or (d) PP2 (0.08, 0.16, and 0.32 µM) at 26 °C for 30 min, or (**c**) genistein (25, 50, and 100 µg/mL) for 1 h. After removing the drugs, the BmN cells were incubated in TC-100 medium containing 10% FBS and BmCPV virions (10^8^ lysed polyhedra/mL, 10 μL). The infected BmN cells were collected at 48 h post-infection. Virus multiplication was estimated by determining the relative expression level of the BmCPV *vp1* gene to *actin A3* by RT-qPCR using the primers pair REVP1-1/REVP1-2. Error bars indicate standard deviations. ***P < 0.001.

**Figure 3:**
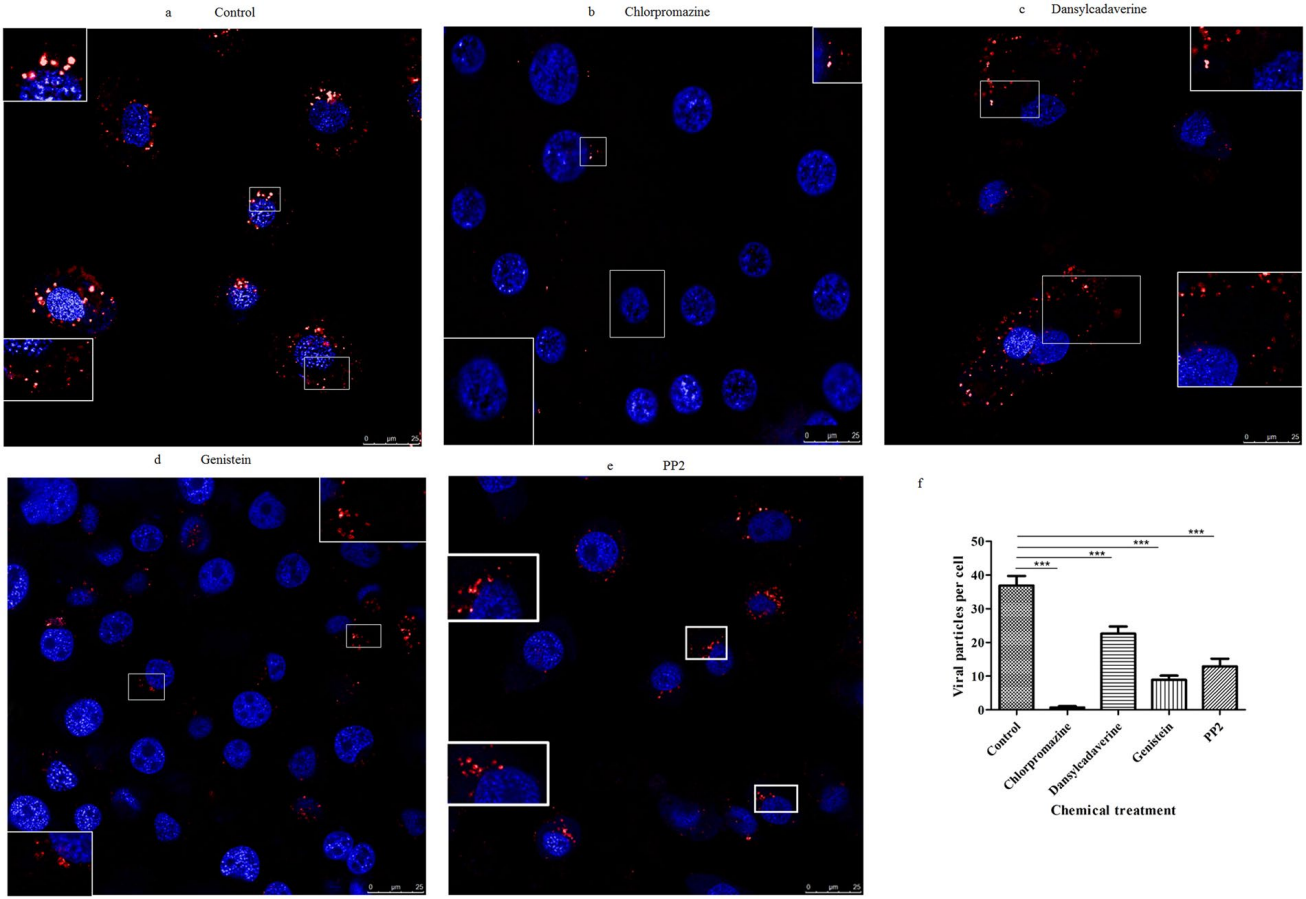
Confocal microscopy observations of BmCPV internalization and different distribution of BmCPV particls with different endocytic inhibitors. BmN cells were treated without inhibitors as a control (**a**); the cells were treated with chlorpromazine (2 mM) (**b**), dansylcadaverine (2 mM) (**c**), genistein (50 μg/mL) (**d**), or PP2 (0.16 μM) (**e**), for 30 min or 1 h, followed by inoculation with A546-labeled BmCPV virions. Thirty minutes after inoculation, images were captured by a confocal microscopy. The nucleus was stained withDAPI. (**f**) Quantification of internalized BmCPV particles in single planes of view (*n* = 21 cells). Error bars indicate standard deviations. ***P < 0.001.

To further understand the functions of endocytosis during the cell entry of BmCPV, we investigated the effects of chlorpromazine, dansylcadaverine, genistein, and PP2 which were the endocytic inhibitors on the entry of BmCPV into BmN cells. Initially, the effect of the inhibitors on the propagation of BmN cells was investigated by a 3-(4,5-dimethylthiazol-2-yl)-2, 5-diphenyltetrazolium bromide (MTT) assay. The results showed that negative effects were not observed when the BmN cells were treated with these different drugs (Fig. S1). Subsequently, we investigated the effect of these inhibitors on the multiplication of BmCPV. As shown in Fig. 2, the relative expression level of the BmCPV *vp1* gene to actin *A*3 gene decreased substantially in the inhibitor-treated BmN cells, compared with that of a control which was not treated with inhibitors. Among the four inhibitors, chlorpromazine was the most effective at inhibiting the infectivity of BmCPV in BmN cells. These data indicated that endocytic inhibitors can suppress the cell infection of  BmCPV. Next, we used confocal microscopy to further examine the effect of these four inhibitors on the internalization of BmCPV into BmN cells, the results showed that the number of BmCPV virions (red fluorescence) in the BmN cells treated with inhibitors was less than that in control cells (Fig. 3a–e), suggesting that the entry of BmCPV into BmN cells was inhibited by endocytic inhibitors. BmCPV particles were observed around the outline of BmN cells that were treated with dansylcadaverine (Fig. 3c), while BmCPV particles in cells treated with genistein or PP2 were mostly in the perinuclear region (Fig. 3d,e), suggesting that the distribution of the internalized BmCPV particles in the cells differed in response to treatments with different endocytic inhibitors. Quantification of the internalized BmCPV particles was conducted according to a previous report^19^ and the results revealed differences in the number of BmCPV particles that were internalized into cells incubated in normal medium or medium containing the inhibitors (Fig. 3f). Low density lipoprotein (LDL) was considered to enter by clathrin-mediated endocytosis^20–22^ Dil-labeled acetylated LDL (Dil-ac-LDL) (Bioquote, York, United Kingdom) was used to assess inhibition of the endocytic inhibitors on endocytosis, the results showed that the number of Dil-ac-LDL marker (red) was decreased in BmN cells that were treated with endocytic inhibitors (Fig. S2), corroborating that clathrin-mediated endocytosis was effectively and specifically blocked under our experimental conditions. Together, the results suggest that clathrin-mediated endocytosis plays an important role in the entry of BmCPV into BmN cells, and that other endocytic pathways also hinder the infection of BmCPV.

### Endocytic inhibitors reduce the infectivity of BmCPV in silkworms

**Figure 4:**
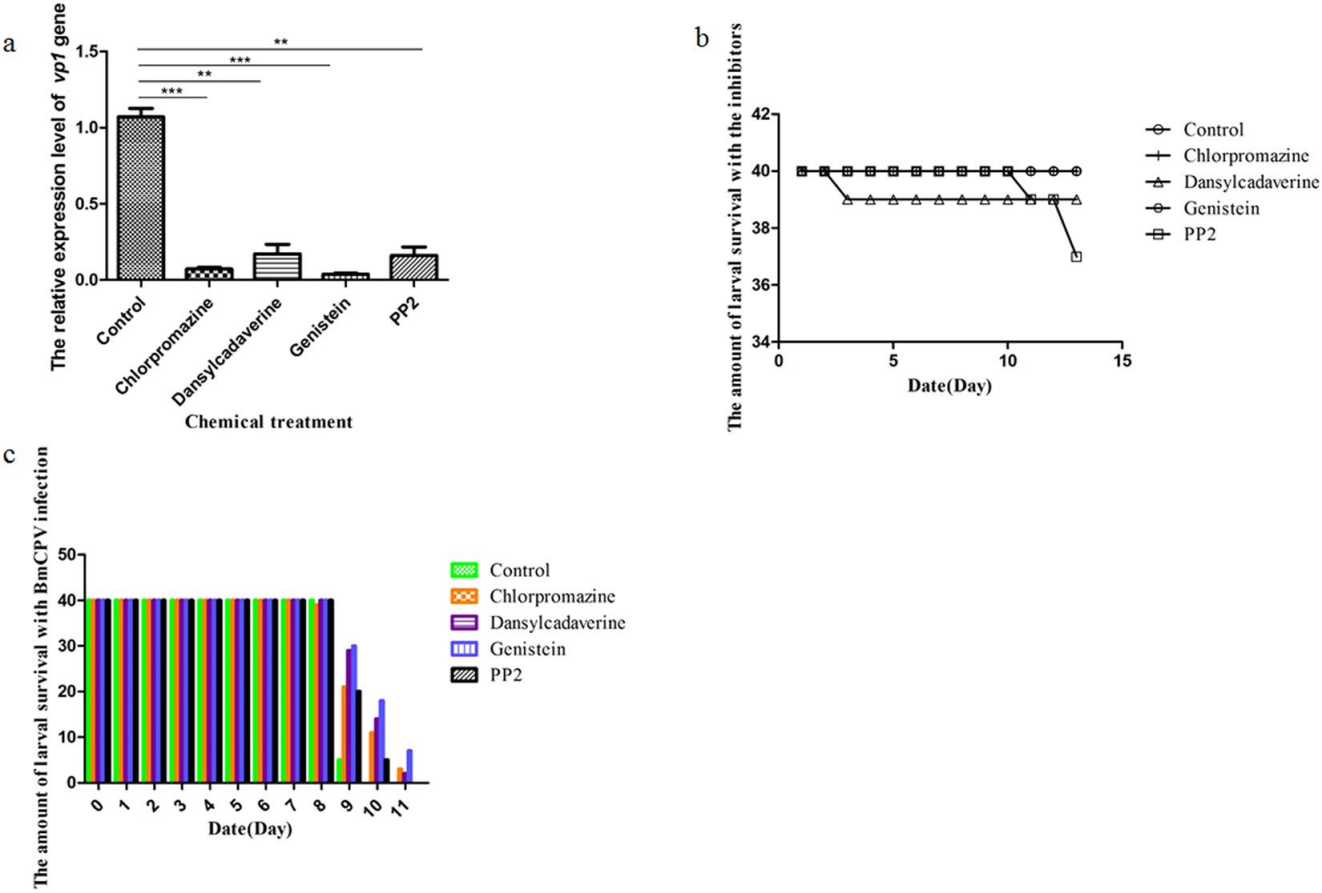
The inhibition effect on BmCPV infection of silkworms with endocytic inhibitors. (**a**) Silkworms were fed mulberry leaves coated with water (control), 1 mM chlorpromazine, 1 mM dansylcadaverine, 0.08 μM PP2, or 25 μg/mL genistein, and then they were infected with BmCPV. The dissected midguts from 15 silkworm larvae at 120 h post-infection were used to isolate total RNA. The relative expression level of the BmCPV *vp1* gene in the silkworm midgut at 120 h post-infection was determined by RT-qPCR. Error bars indicate standard deviations. *P < 0.5; **P < 0.01. (**b**) Silkworms were fed mulberry leaves coated with water (control), 1 mM chlorpromazine, 1 mMdansylcadaverine, 0.08 μM PP2, or 25 μg/mL genistein, and the survival of the silkworms with endocytic inhibitors was calculated to test the endocytic inhibitors’ toxicity in 13 days. (**c**) Silkworms were fed mulberry leaves coated with water (control), 1 mM chlorpromazine, 1 mMdansylcadaverine, 0.08 μM PP2, or 25 μg/mL genistein, and then they were infected with BmCPV. The amount of larval survival with BmCPV infection was calculated after 11 days.

We further investigated whether the endocytic inhibitors could depress the infectivity of BmCPV in silkworms. The relative expression level of the BmCPV*vp1* gene to actin *A*3 gene in the midgut of silkworms that were treated with chlorpromazine, dansylcadaverine, genistein, and PP2 was reduced by 81.01%, 52.94%, 78.65%, and 46.82%, respectively, at 120 h post-infection compared with control silkworms (Fig. 4a), and the cultured silkworms survived for a dozen days in the presence of the inhibitors when they were supplied with sufficient food and a proper temperature (Fig. 4b). The onset of silkworm cytoplasmic polyhedrosis was delayed and the incidence was reduced by oral administration of inhibitors (Fig. 4c). These results suggested that the endocytic inhibitors may be candidate drugs for treating silkworm cytoplasmic polyhedrosis.

### Silencing the *AP-1* and *clathrin* genes decreases the infectivity of BmCPV in BmN cells

**Figure 5:**
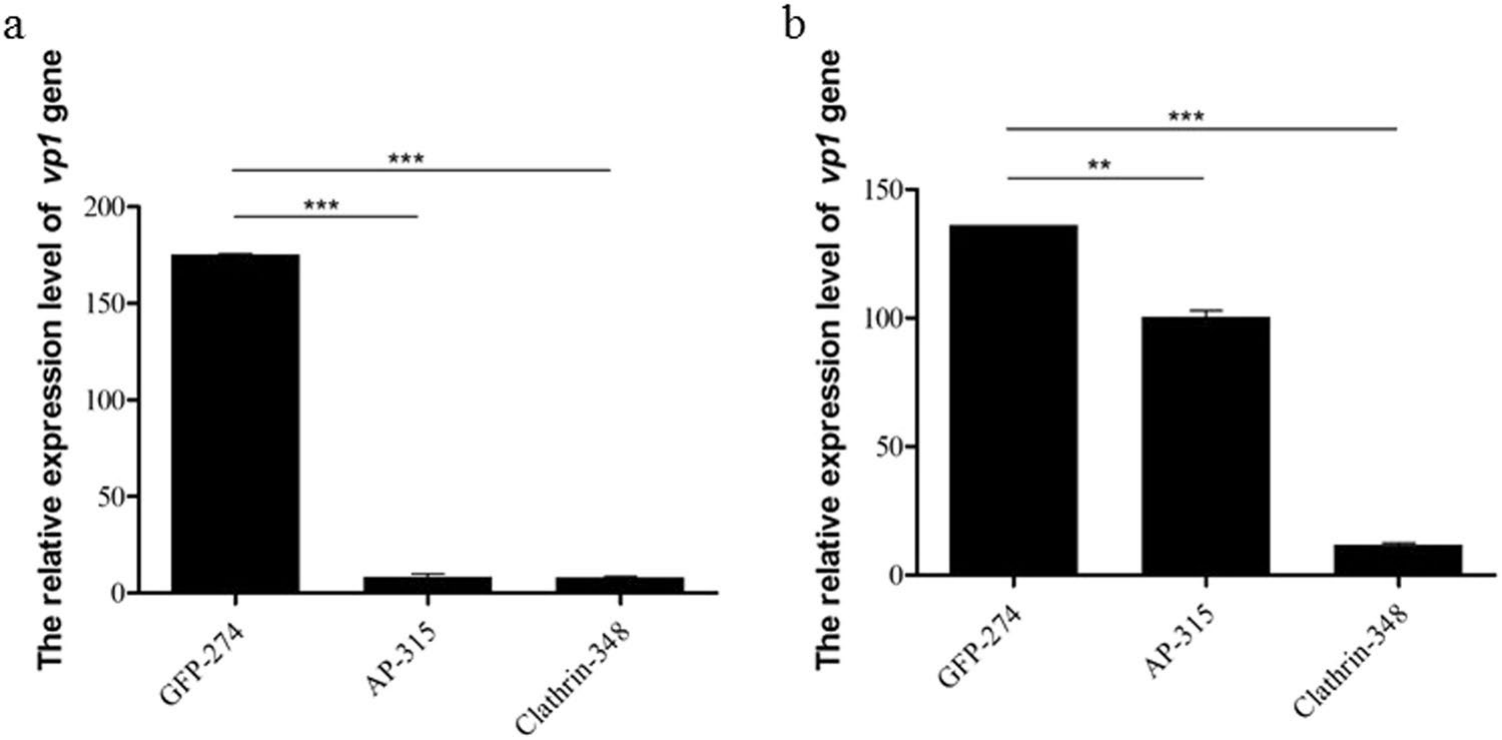
Effect of silencing the *AP-1* and *clathrin* genes on the BmCPV infection of BmN cells and silkworms. (**a**) The relative expression level of the BmCPV *vp1* gene in BmN cells treated with AP-315 or clathrin-348 siRNAs at 48 h post-inoculation. (**b**) The relative expression level of the BmCPV *vp1* gene in the midguts of silkworms injected with the AP-315 or clathrin-348 siRNAs at 48 h p.i. Error bars indicate standard deviations. *P < 0.5; **P < 0.01; ***P < 0.001.

To further understand the role of clathrin-mediated endocytosis in the cell entry of BmCPV, both the adaptor protein complex-1 gamma subunit AP-1 (AP-1, GenBank accession No. JQ824201.1) and clathrin heavy chain (clathrin, GenBank accession No. NM_001142971.1) proteins that were interacting proteins of BmCPV were chosen^23^ and the effects of silencing these genes on the infectivity of BmCPV in BmN cells were investigated. Quantitative reverse transcription–polymerase chain reaction (RT-qPCR) results showed that AP-315 and clathrin-348 were specific small interfering RNAs (siRNAs) for the *AP-1* and *clathrin* genes (Fig. S3a,b), the relative expression levels of the *AP*-1 and *clathrin* genes in the BmN cells decreased by 72.20% and 76.50% at 48 h post-transfection with AP-315 and clathrin-348 siRNAs, respectively. Western blotting further confirmed that the levels of AP-1 and clathrin proteins in the BmN cells decreased (Fig. S3e). Then, BmN cells that were transfected with a siRNA (either AP-315 or clathrin-348) were infected with BmCPV, and the relative expression level of the BmCPV *vp1* gene was determined by RT-qPCR. The results showed that the relative expression level of the BmCPV *vp1* gene decreased by 94.35% and 95.16% after silencing the *AP-1* and *clathrin* genes (Fig. 5a), respectively, compared with the control (an siRNA targeting the green fluorescent protein GFP encoding gene). Similar results were also found in silkworms, as the relative expression levels of the *AP-1* and *clathrin* genes in the silkworm midgut decreased by 24.28% and 90.80% at 48 h post-injection of the AP-315 and clathrin-348 siRNAs, respectively, into the silkworms’ hemolymph (Fig. S3c,d), while the relative expression level of the BmCPV *vp1* gene decreased by 24.49% and 90.78%, respectively (Fig. 5b). As a whole, the inhibition in *vp*1 expression of the siRNAs (AP-315 and clathrin-348) positively correlated with silencing efficacy of genes (*AP-1* and *clathrin*). These results indicated that the infection of host cells with BmCPV could be depressed by silencing *AP-1* and *clathrin* genes.

### Abolishing the functions of clathrin and AP-1 with antibodies reduces BmCPV infectivity in BmN cells

**Figure 6:**
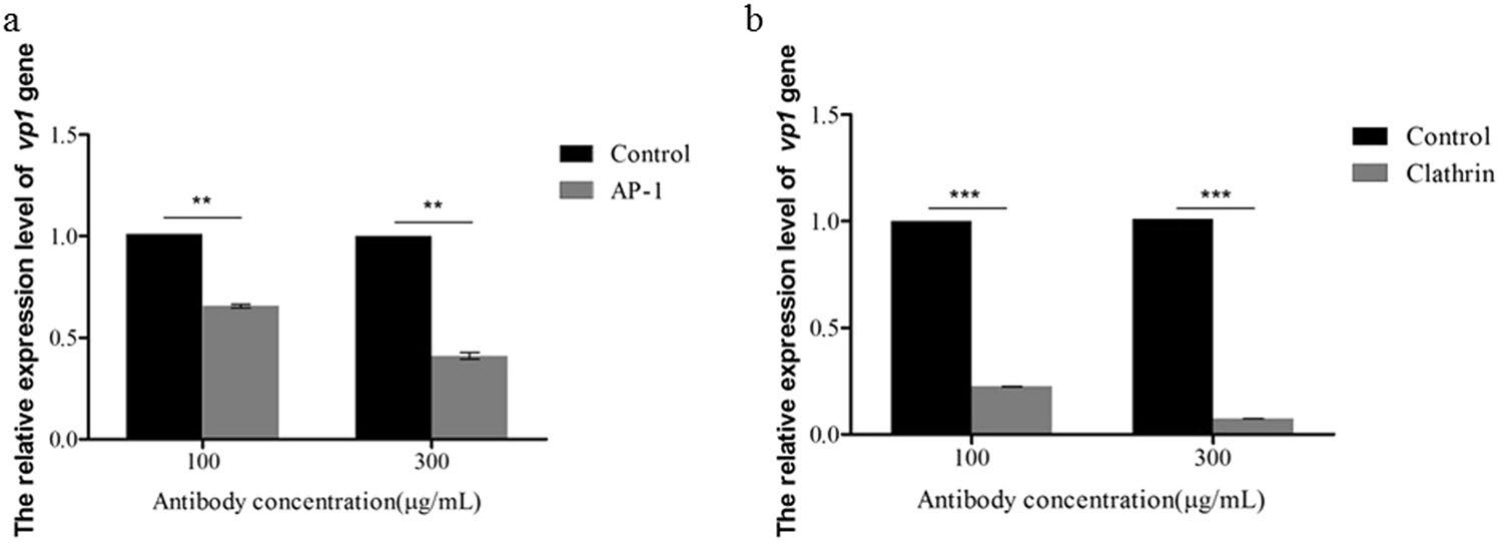
The descreased infection of BmN cells by BmCPV with antibodies to block AP-1 and clathrin proteins. (**a** and **b**) BmN cells were treated with either anti-AP-1 or anti-clathrin antibodies at final concentrations of 100 and 300 μg/mL, respectively, for 1 h. Then, the treated BmN cells were infected with BmCPV, and the relative expression level of the BmCPV *vp1* gene at 48 h post-infection was determined by RT-qPCR; control, the cells were treated with non-immune mouse serum; AP and clathrin, the cells were respectively treated with anti-AP-1 and anti-clathrin; the *actin A3* housekeeping gene was used as an internal reference. Error bars indicate standard deviations. **P < 0.01; ***P < 0.001.

We generated mouse polyclonal antibodies against AP-1 and clathrin, and we detected the blocking effects of these antibodies by confocal microscopy (Fig. S4). The results showed that the antibodies entered BmN cells and abolished the functions of AP-1 and clathrin. Then, we used these antibodies to assess the effects of abolishing the functions of clathrin and AP-1 on BmCPV infectivity of BmN cells. The results showed that the relative expression level of the BmCPV *vp1* gene in BmN cells treated with the anti-AP-1 antibody was reduced by 33.33–57.12% and by 77.28–92.57% with the anti-clathrin antibody at 48 h post-infection, compared with the control that was treated with non-immune mouse serum (Fig. 6a,b). Moreover, we also found that the number of Dil-ac-LDL marker was decreased in these antibodies-treated BmN cells, corroborating that clathrin-mediated endocytosis were blocked under our experimental conditions (Fig. S5). The results suggest that the entry of BmCPV into BmN cells could be inhibited by abolishing the functions of proteins that are involved in clathrin-mediated endocytosis.

### Genistein and PP2 increase the number of BmCPV virions in lysosomes

**Figure 7:**
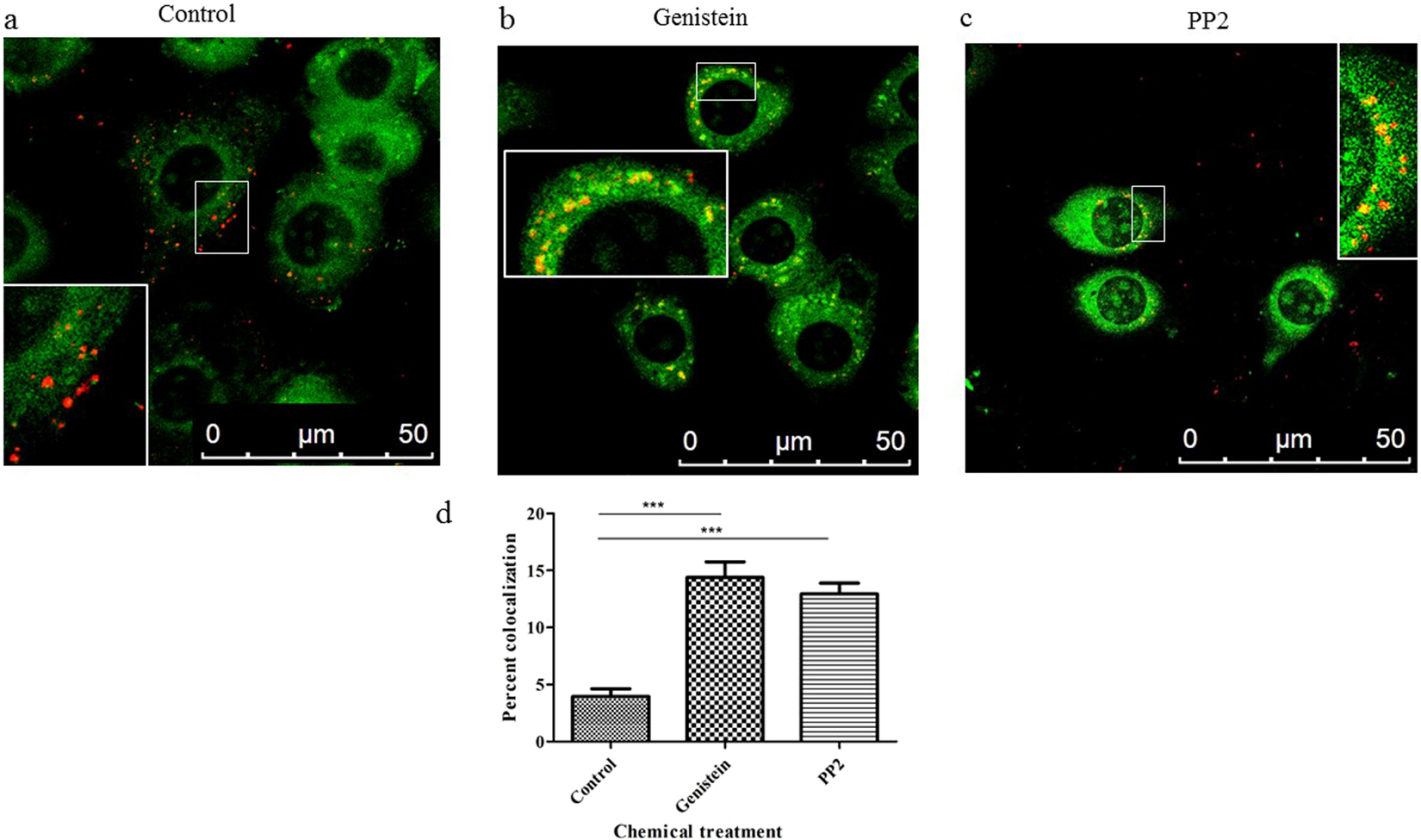
Genistein and PP2 route BmCPV to the wrong destination- lysosomes. (**a**) Normal BmN cells (control) were incubated with A546-labeled BmCPV virions and a lysosomal staining agent for 3 h; (**b**) BmN cells were treated with PP2 (0.16 μM) for 30 min, followed by incubation with A546-labeled BmCPV virions and a lysosomal staining agent for 3 h; (**c**) BmN cells were treated with genistein (50 μg/mL) for 1 h, followed by incubation with A546-labeled BmCPV virions and a lysosomal staining agent for 3 h; (**d**) quantification of the spectral overlap of BmCPV particles with lysosomes. The spectral overlap is presented as the percent co-localization (*n* = 21 cells). Error bars indicate standard deviations. ***P < 0.001.

To determine whether genistein or PP2 alters the distribution of the internalized BmCPV particles, we used confocal microscopy to locate internalized BmCPV virions and lysosomes. The results showed that most of the internalized BmCPV virions (red) were not distributed in lysosomes (green) in control cells (cells that were not incubated with either genistein or PP2) (Fig. 7a). In contrast, in cells that were incubated with either genistein (Fig. 7b) or PP2 (Fig. 7c), the distribution of BmCPV virions to lysosomes increased. The pixel intensity profile of lysosome-positive areas (green) of the control cells displayed little overlap with the profile of the BmCPV particles (red) (Fig. 7a). In contrast, cells that were incubated with either genistein (Fig. 7b) or PP2 (Fig. 7c) exhibited an obvious overlap between the BmCPV and lysosome spectra. Quantification of the spectral overlap of the BmCPV particles with lysosomes revealed an increased co-localization of BmCPV particles with a lysosomal marker in cells that were incubated with either genistein or PP2, compared with the control (Fig. 7d). These results indicate that genistein and PP2 increase the number of BmCPV virions in lysosomes, which is a nonproductive pathway of viral entry.

Leupeptin is a lysosomal inhibitor, and we assessed whether it could rescue the BmCPV infectivity of cells that were pre-incubated with either genistein or PP2. The results showed that the leupeptin did not affect the amount of the BmCPV (red) entered into cells (Fig. 6a,d,g), suggesting that leupeptin could not help more BmCPVs enter into the cells. The number of BmCPV particles (red) in control cells (Fig. S6a) was greater than that in the PP2-treated (Fig. S6b) and genistein-treated (Fig. S6c) cells. However, the number of BmCPV particles (red) in the PP2-treated (Fig. S6e) and genistein-treated (Fig. S6f) cells that were pre-incubated with leupeptin hemisulfate was greater than that in the corresponding control cells that were not pre-incubated with leupeptin. Quantification of the internalized BmCPV particles revealed obvious differences in the number of internalized BmCPVparticles between the PP2 (or genistein)-treated cells and the PP2 (or genistein)-treated cells that were pre-incubated with leupeptin (Fig. S6g). Consequently, genistein and PP2 led to aberrant transport of BmCPV particles to lysosomes; the lysosomal enzymes were inhibited in the leupeptin-treated cells, more fluorescent dye-labeled BmCPV particles were observed in the PP2 (or genistein)-treated cells that were pre-incubated with leupeptin because they were not digested.

## Discussion

Silkworm cytoplasmic polyhedrosis is caused by BmCPV infection. To date, there are no clear mechanisms to describe the entry of BmCPV into cells. In the present study, we studied the route of entry of BmCPV into BmN cells, as well as the effect of blocking the entry pathway with endocytic inhibitors on BmCPV infectivity. The results showed that BmCPV virions might be internalized into cells by endocytosis.

Viruses can be internalized into cells by a variety of endocytic pathways, including clathrin-mediated endocytosis, caveolar endocytosis, and clathrin- and caveolae-independent endocytosis. In addition, some viruses enter cells through direct fusion with the plasma membrane^11^. It has been shown recently that rotaviruses, which are members of the family *Reoviridae*, enter cells by endocytosis, and that different rotavirus strains use different endocytic pathways^24^. Most rotaviruses enter cells by clathrin-mediated endocytosis. In contrast, a simian rhesus rotavirus strain follows an endocytic pathway that is independent of clathrin and caveolin, but dependent on the presence of dynamin 2, the small GTPases RhoA and Cdc42, actinin-4, and cholesterol on the cell surface^25^ suggesting that rotaviruses have the flexibility to preferentially use one route of entry or that the entry factors for rotaviruses are superfluous. To date, the routes of entry of viruses into cells are still poorly understood, particularly for insect reoviruses, including BmCPV, which also belongs to the *Reoviridae* family.

In this study, we observed the initiating event for cell entry of BmCPV by electron microscopy, and the results showed that BmCPV virions first attached to the plasma membrane, and then they were internalized into cells via plasma membrane invagination. Subsequently, the viral particles were delivered to an endosome-like structure. These results strongly suggest that BmCPV cell entry is mediated by endocytosis.

To date, pharmacological inhibitors of different endocytic pathways have been used extensively to demonstrate the receptor-mediated endocytosis of viruses^26,13,27^. Therefore, we used dansylcadaverine, chlorpromazine, genistein, and PP2, which were endocytic inhibitors, to study the entry of BmCPV into BmN cells. In the present study, the relative expression level of the BmCPV *vp1* gene in BmN cells that were treated with dansylcadaverine decreased in a dose-dependent manner compared with the control, suggesting that BmCPV entry into host cells via a receptor-mediated endocytic pathway. And we also found that the relative expression level of the BmCPV *vp1* gene obviously decreased when BmN cells were treated with chlorpromazine, suggesting that BmCPV entry into host cells might be mediated by clathrin-dependent endocytosis.

Viruses labeled with fluorescent dyes have been used widely to study the cell entry, transport and distribution of viruses^28^. The confocal fluorescence microscope observations showed that the numbers of internalized BmCPV virions in chlorpromazine- treated cells were less than that in control cells, suggesting that the cell entry of BmCPV was inhibited. This result further confirmed that clathrin-mediated endocytosis plays an important role in BmCPV internalization.

Various types of endocytosis involve different proteins. Clathrin plays a major role in the formation of clathrin-coated vesicles^29^ and AP-1 was proposed to interact with the scaffolding protein clathrin^30^. APs can form a heterotetrameric complex that is involved in binding to the target membrane and mediating interactions between clathrin and other cargo adaptors^31^ therefore, both AP-1 and clathrin are involved in the clathrin-dependent endocytic pathway. Our previous study suggested that BmCPV is internalized by clathrin-mediated endocytosis utilizing a β1 integrin-dependent process, and found that both the AP-1 and clathrin could interact with BmCPV^23^. The blockade of receptors located on the surface of specific cells by antibodies can significantly reduce viral infections^32,33^ thus, we studied the effects of silencing the *AP-1* and *clathrin* genes, and abolishing the functions of clathrin and AP-1 with antibodies on BmCPV infectivity. The relative expression levels of the BmCPV *vp1* gene decreased following siRNA-mediated inhibition of the *AP-1* or *clathrin* genes, as well as the antibody-mediated blockade of AP-1 or clathrin, these results also provided evidence that the entry of BmCPV into BmN cells relies upon AP-1 and clathrin, and they further indicated that BmCPV internalization is mediated by the clathrin-mediated endocytic route.

After virions are internalized, efficient infection depends on their escape from endocytic vesicles, and delivery of the viral genome^34^. Endocytosed reoviruses undergo stepwise disassembly, which is catalyzed by cathepsin proteases, followed by endosomal membrane penetration and delivery of transcriptionally active core particles into the cytoplasm^28,35,36^. In the present study, we found that the distribution of virions in lysosomes was increased in BmN cells that were treated with genistein or PP2, which confirmed that both genistein and PP2 lead to a nonproductive BmCPV infection by aberrantly transporting virions to lysosomes, suggesting that Src kinase-mediated, productive endocytic sorting of BmCPV occurs during cell entry.

Lysosomes are acidic and contain cathepsins, the endocytosed reoviruses can be digested in lysosomes^37^. Leupeptin is a reversible inhibitor of serine and cysteine proteases, and it inhibits trypsin, papain, and cathepsin B^38^. In the present study, the results showed that leupeptin increased the number of productive BmCPV virions, which suggested that lysosomes disassemble BmCPV virions, thereby decreasing the infectivity of BmCPV in BmN cells. These results suggest that targeting BmCPV particles to endocytic organelles for viral disassembly is an effective way of preventing silkworm cytoplasmic polyhedrosis.

To date, sericulture is often threatened by cytoplasmic polyhedrosis, but there are no effective medicines for the prevention and treatment of this disease. Inhibiting the cell entry of virus plays an important role in the prevention and cure of virus diseases. Our results suggested that the entry of BmCPV into cells was dependent on clathrin-mediated endocytosis, suggesting that every step of the clathrin-mediated endocytosis pathway can be regard as a potential therapeutic target for silkworm cytoplasmic polyhedrosis. In present study, the effects of blocking the entry pathway with endocytic inhibitors (dansylcadaverine, chlorpromazine, genistein, and PP2) on BmCPV infectivity were studied. We found that the relative expression level of the BmCPV *vp1* gene decreased in the cultured cells that were treated with the inhibitors, similar results were also found in the midgut of BmCPV-infected silkworms that were treated with the inhibitors. Moreover, the progression of silkworm cytoplasmic polyhedrosis was delayed and the incidence was reduced by oral administration of inhibitors. These findings provided a clue to screen for therapeutic compounds that prevent cytoplasmic polyhedrosis.

## Materials and Methods

BmN cells, which are derived from silkworm ovarian tissue, were grown in TC-100 medium (AppliChem, Darmstadt, Germany) containing 10% fetal bovine serum (FBS).

### BmCPVvirion preparation

Newly exuviated third-instar larvae (the Dazao strain) were fed BmCPV-polyhedra-coated mulberry (*Morus*) leaves (containing 10^8^ polyhedra per mL) for 8 h. The purified polyhedra from the infected midgut were resuspended in 0.09 mol/L Na_2_CO_3_–0.01 mol/L NaHCO_3_ buffer at 37 °C for 10 min, followed by adjusting the pH to 8.0 with 1 M HCl, and the supernatant was used as the BmCPV virions stock after centrifugation for 10 min at 12,000 × *g*.

### Electron microscopy

To observe the entry route of BmCPV into cells, 2 × 10^5^ BmN cells were incubated with 1 mL of serum-containing medium and BmCPV (10^10^ lysed polyhedra/mL, 10 μL). The BmN cells were collected at 0.5 h and 1.5 h post-incubation, and they were examined with a transmission electron microscope (Hitachi-H7650, Tokyo, Japan) according to our previous report^39^.

### Transfection of siRNAs

To understand the cell entry of BmCPV, the effect of silencing the *clathrin* and *AP-1* genes, which are involved in clathrin-mediated endocytosis, was investigated using RNA interference. Briefly, 1 μg of a specific siRNA targeting either the *AP-1* or *clathrin* genes (Table S1), which was synthesized by the Gima Corporation (Shanghai, China), was transfected into 2 × 10^5^ BmN cells using Lipofectamine (Roche Diagnostics, Mannheim, Germany). Simultaneously, a siRNA targeting the GFP gene was used a negative control. The relative gene expression levels at 48 h post-transfection with the siRNAs were estimated by RT-qPCR using the primer pairs REAP-1-1/REAP-1-2 and RECLAT-1/RECLAT-2 (Table S2). Furthermore, the levels of the clathrin and AP-1 proteins in BmN cells at 48 h post-treatment with the clathrin-348 siRNA and the AP-315 siRNA were assessed by Western blotting with their corresponding antibodies, respectively. The tublin was used as an internal reference and the images of the western blot were gotton by multifunction analysis imaging system ChemiScope6300 (Clinx Science Instruments Co., Ltd, Shanghai, China).

BmN cells were infected with BmCPV (10^8^ lysed polyhedra/mL, 10 μL), and the cells were collected at 48 h post-infection. Virus multiplication was estimated by determining the relative expression of the BmCPV structural protein-encoding *vp1* gene by RT-qPCR using the primer pair REVP1-1/REVP1-2 (Table S2).

Total RNA was isolated from the collected BmN cells and silkworm midguts. The relative expression level was quantified by RT-qPCR using an RT-qPCR kit (TransGenBiotech, Beijing, China) according to the manufacturer’s protocol using the primers listed in Table S2.

The RT-qPCR experimets were performed by relative quantitative PCR, and the *B. mori actin3* housekeeping gene was used as an internal reference to obtain the relative expression of the target genes. The relative expression of the target genes was calculated by the 2^−ΔΔCt^ method^40^. The experiment was performed with three replicates.

Larvae of fourth-instar silkworms (strain Dazao) were injected with 10 μL of the mixture of siRNA (1 μg) and Lipofectamine (1 μL), and the relative expression levels of the *clathrin* and *AP-1* genes in the silkworm midgut were analyzed by RT-qPCRat 48 h post-injection. Moreover, at 48 h post-injection, the larvae were fed mulberry leaves coated with BmCPV polyhedra (10^8^ polyhedra per mL) for 8 h. The dissected midguts from 10 silkworm larvae at 48 h post-infection were used to isolate total RNA. Virus multiplication was estimated by determining the relative expression of the BmCPV*vp1* gene by RT-qPCR.

### Blocking of BmCPV infectivity with antibodies against clathrin and AP-1

The membrane-spanning regions and orientations of the clathrin heavy chain (GenBank accession No. NP_001136443.1) and AP-1 (GenBank accession No. AFN25815.1) proteins were predicted using the TMpred program (https://embnet.vital-it.ch/software/TMPRED_form.html). The results showed that three and one putative transmembrane helices were found in silkworm clathrin heavy chain and AP-1, respectively. Thus, an antibody blockade assay could be an effective strategy for assessing the functions of AP-1 and clathrin^32,33^.

To confirm entry of AP-1 and clathrin-specific antibodies into the BmN cells, the BmN cells were respectively treated with normal mouse serum, anti-AP-1 antibody, and anti-clathrin antibody at final concentration of 1000 μg/mL for 1 h, and the treated cells were washed with 1× phosphate-buffered saline (PBS) and fixed with 4% paraformaldehyde for 5 min. Subsequently, the immunofluorescent staining was followed and observed under a confocal microscope.

BmN cells (2 × 10^5^) were incubated with anti-AP-1 or anti-clathrin antibodies (at final concentrations of 100 μg/mL and 300 μg/mL, respectively) at room temperature for 1 h; non-immune mouse serum was used as a control. The treated cells were infected with 10 μL of BmCPV (10^8^ lysed polyhedra/mL) after washing three times with 1× PBS. The infected cells were collected at 48 h post-infection, and the relative expression level of the BmCPV *vp1* gene was determined by RT-qPCR using the primer pair REVP1-1/REVP1-2 (Table S2) to estimate BmCPV multiplication in the cells.

### Treatment of BmN cells and silkworm larvae with endocytic inhibitors

BmN cells (2 × 10^5^) were incubated with TC-100 medium containing dansylcadaverine (at final concentrations of 1, 2, and 4 mM), chlorpromazine (at final concentrations of 1, 2, and 4 mM), or PP2 (at final concentrations of 0.08, 0.16, and 0.32 µM) at 26 °C for 30 min, or genistein (at final concentrations of 25, 50, and 100 µg/mL) at 26 °C for 1 h^14,16^ and cells that were not treated with the endocytic inhibitors were used as a control. After the BmN cells were incubated in 1 mL of serum-containing medium containing BmCPV (10^8^ lysed polyhedra/mL, 10 μL), the infected BmN cells were collected at 48 h post-infection, and then the relative expression level of the BmCPV *vp1* gene was determined by RT-qPCR using the primer pair REVP1-1/REVP1-2 (Table S2) to estimate BmCPV multiplication in the cells.

Inhibitors of endocytosis can inhibit cell entry of viruses^14–16,19^ therefore, dansylcadaverine, chlorpromazine, genistein, and PP2 can be used for developing prevent and treatment drugs for *B. mori* cytoplasmic polyhedrosis. To assess control efficiency of the inhibitors on cytoplasmic polyhedrosis, the larvae of second-instar silkworms (strain Jingsong × Haoyue) were fed mulberry leaves that were coated with double-distilled water (control), dansylcadaverine (1 mM), chlorpromazine (1 mM), PP2 (0.08 μM), or genistein (25 μg/mL) for 8 h, and then they were fed mulberry leaves coated with BmCPV polyhedra (10^8^ polyhedra per mL) for 8 h following previous study^41^. The dissected midguts from 15 silkworm larvae at 120 h post-infection were used to isolate total RNA. Virus multiplication was estimated by determining the expression of the BmCPV *vp1* gene by RT-qPCR using the primer pair REVP1-1/REVP1-2 (Table S2). The experiments were performed three times. The survival of the larvae that were treated with the inhibitors was determined, and the morbidity induced by BmCPV was calculated as the number of surviving BmCPV-infected larvae/the total number of surviving larvae.

### Production of succinimidyl ester Alexa Fluor 546-labeled BmCPVvirions

BmCPV virions were purified from polyhedral according to our previous study^23^. To track the entry route of BmCPV into cells, 15 mg of the purified polyhedra was suspended in 1 ml of 0.09 mol/L Na_2_CO_3_–0.01 mol/L NaHCO_3_ buffer at 37 °C for 10 min, followed by adjusting the pH to 8.0 with 1 M HCl, after centrifugation for 3 h at 60,000 × g, the precipitate was dissolved in 1 mL of 0.05 M sodium bicarbonate (pH 8.5) with 50 μM succinimidyl ester A546 (Life Technologies, Carlsbad, CA, USA) and incubated at room temperature for 90 min in the dark^28^ and then they were dialyzed against 1× PBS at 4 °C overnight and stored at 4 °C.

### Location of BmCPV particles following treatment with the endocytic inhibitors

BmN cells (2 × 10^5^) were treated with TC-100 medium containing dansylcadaverine (2 mM), chlorpromazine (2 mM), or PP2 (0.16 μM) for 30 min, or genistein (50 μg/mL) for 1 h; cells that were not treated with the endocytic inhibitors were used as a control. The cells were washed three times with PBS to remove the inhibitors, and then they were incubated in 1 mL of TC-100 medium containing 10% FBS and 15 μL of A546-labeled BmCPVvirions (45 μg/mL) for 30 min. The treated BmN cells were again washed three times with PBS and fixed with 4% paraformaldehyde for 5 min. Subsequently, immunofluorescent staining was performed, and internalized virions were quantified by enumerating viral particles that were labeled with A546.

### Colocalization of BmCPV particles and lysosomes

BmN cells (2 × 10^5^) were plated in six-well plates with a coverslip and incubated with TC-100 medium containing genistein (at a final concentration of 50 µg/mL) for 1 h or PP2 (at a final concentration of 0.16 µM) for 30 min at 26 °C; cells that were not treated with the endocytic inhibitors were used as a control. After washing three times with pre-cooled PBS, the BmN cells were cultured in 1 mL of TC-100 medium containing 10% FBS, 15 μL of A546-labeled BmCPVvirions (45 μg/ml), and 1 mL of lysosomal staining agent (Lysosomal Staining Kit *Green Fluorescence with 405 nm Excitation, AAT Bioquest Inc., Sunnyvale, CA, USA) for 3 h at 26 °C. Subsequently, the treated BmN cells were examined by a confocal microscopy. Images were captured using an A1 confocal microscope (Nikon Instruments Inc., Melville, NY, USA). The colocalization of lysosomes and BmCPV virions was quantified by enumerating viral particles that were labeled with A546.

### Statistical analysis

Mean values for at least three replicates were compared using an unpaired *t*-test (GraphPad Prism). *P* values < 0.05 were considered to be statistically significant.

## Electronic supplementary material

Supplemenary materials
