# Supplementary material for: Clathrin-mediated endocytosis is a candidate entry sorting mechanism for Bombyx mori cypovirus
Source: Sci Rep. 2018 May 8;8:7268. doi: 10.1038/s41598-018-25677-1 (PMC5940776; doi:10.1038/s41598-018-25677-1)
Supplement: Supplementary file 1 — Supplemenary materials [file 41598_2018_25677_MOESM1_ESM.doc]

Supplementary Material Files

Clathrin-mediated endocytosis is a candidate entry sorting mechanism for *Bombyx mori* cypovirus

Fei Chen ^1^*, Liyuan Zhu^1^*, Yiling Zhang^1^*, Dhiraj Kumar^1^, Guangli Cao^1,2^, Xiaolong Hu^1,2^, Zi Liang^1^, Sulan Kuang^1^, Renyu Xue^1,2^, Chengliang Gong^#^ ^1,2^

1: School of Biology & Basic Medical Science, Soochow University, Suzhou 215123, China

2: National Engineering Laboratory for Modern Silk, Soochow University, Suzhou 215123, China

*These authors contributed equally to this work

# Corresponding author

Tel.: +86-521-65880183; Fax: +86-521-65880183

E-mail addresses: gongcl@suda.edu.cn

**Materials and methods**

**Preparation of polyclonal antibodies**

To clone the partial sequences of the *AP-1* (nt 272–672 in sequence JQ824201.1) and *clathrin* *heavy chain* (nt 515–1216 in sequence NM_001142971.1) genes, the PCR was conducted using primers (Table S3). The PCR products were respectively cloned into a prokaryotic expression vector pET28a (+) (Novagen, Darmstadt, Germany) to generate recombinant vectors. The recombinant proteins with a histidine tag were expressed in *Escherichia coli* strain BL21. Western blotting was performed using a mouse anti-His6 antibody (Tiangen, Beijing, China) as the primary antibody and horseradish peroxidase-conjugated goat anti-mouse IgG (Tiangen, Beijing, China) as the secondary antibody. Recombinant proteins recovered from the gel were used to immunize Kunming mice (Soochow University, Suzhou , China) by subcutaneous injection to prepare anti-AP-1 and anti-clathrin antibodies.

**3-(4, 5-dimethylthiazol-2-yl)-2, 5-diphenyltetrazolium bromide (MTT) assay**

To estimate the cytotoxicity of drugs to BmN cells, genistein (Sigma–Aldrich, St. Louis, MO, USA) and PP2 (Sigma-Aldrich) were respectively dissolved in dimethyl sulfoxide according to the manufacturer’s instructions. Chlorpromazine and dansylcadaverine (both from Sigma-Aldrich) were respectively dissolved in double-distilled water and alcohol. BmN cells (2×10^3^) were cultured in TC-100 medium containing 10 % FBS for 24 h at 26 °C. Then, the cells were incubated with TC-100 medium respectively containing chlorpromazine (1, 2 and 4 mM), dansylcadaverine (1, 2, and 4 mM), and PP2 (0.08, 0.16, and 0.32 µM) at 26 °C for 30 min, and genistein (25, 50, and 100 µg/ml) at 26 °C for 1 h. After washing three times with 1× phosphate-buffered saline (PBS), the cells were cultured in normal medium for 24 h. Subsequently, 20 μl of MTT reagent (Biosharp, Shanghai, China) was added into the medium, and the BmN cells were incubated for 4 h at 26 °C. After the medium was removed, 150 μl of dimethyl sulfoxide (Chenguang Chemical Co., Ltd., Wuxi, China) was added per well. The optical density at 570 nm was measured with a microplate reader (Multiskan FC, Thermo Fisher Scientific, Waltham, MA, USA). Cells that were not treated with the endocytic inhibitors were used as a control. The experiment was repeated five times.

**Confocal microscopy observations of endocytosed Dil-ac-LDL in the inhibitor-treated BmN cells**

BmN cells were treated with chlorpromazine (2 mM), dansylcadaverine (2 mM), or PP2 (0.16 μM) for 30 min, or genistein (50 μg/mL) for 1 h, followed by incubation with10 μg/mL Dil-labeled acetylated (Dil-ac-LDL) (Bioquote, York, United Kingdom); untreated cells were used as a control. Two hours after incubation, the cells were washed by PBS for 3 times. Then the cells were incubated with 5 μM Dio (Bioquote, York, United Kingdom) for 10 mins, images were captured by a confocal microscopy (Leica, Wetzlar, Germany). The nucleus was stained with 4', 6-diamidino-2-phenylindole (DAPI).

**Confocal microscopy observations of endocytosed Dil-ac-LDL in the antibody-treated BmN cells**

BmN cells were respectively treated with normal mouse serum (control), anti-AP-1 antibody, and anti-clathrin antibody at final concentration of 300 μg/mL for 1 h, followed by incubation with10 μg/mL Dil-ac-LDL for 2 h. Then the cells were washed by PBS for 3 times and were incubated with 5 μM Dio for 10 mins, images were captured by a confocal microscopy. The nucleus was stained with DAPI (1:3,000 dilution).

**Lysosomal inhibitor leupeptin hemisulfate rescues the BmCPV infectivity**

BmN cells (2×10^5^) were plated in six-well plates with a coverslip and incubated in TC-100 medium containing leupeptin hemisulfate (Selleck, Shanghai, China) (at a final concentration of 50 µM) at 26 °C for 30 min; cells that were not treated with leupeptin were used as a control. After washing three times with PBS, the cells were incubated in medium respectively containing genistein (50 µg/ml) and PP2 (0.16 µM) for 30 min at 26 °C. After washing with PBS, the BmN cells were incubated in 1 ml of TC-100 medium containing 10 % FBS, 15 μL of A546-labeled BmCPV virions (45 μg/ml), and 1 ml of lysosomal staining agent for 3 h at 26 °C. After fixation with 4 % paraformaldehyde for 5 min and staining with DAPI, the cells were observed under a confocal microscope. Internalized virions were quantified by enumerating viral particles that were labeled with A546.

**Figure Legends**

**Fig. S1. Effects of endocytic inhibitors on the proliferation of BmN cells.**

BmN cells (2×10^3^) were incubated in TC-100 medium respectively containing chlorpromazine (1, 2, and 4 mM), dansylcadaverine (1, 2, and 4 mM), and PP2 (0.08, 0.16, and 0.32 µM) at 26 °C for 30 min, and genistein (25, 50, and 100 µg/ml) for 1 h. After removing the drugs, the BmN cells were incubated in TC-100 containing 10 % FBS for 24 h. The proliferation of BmN cells was determined by the MTT method. Error bars indicated standard deviations. The optical density at 570 nm was measured with a Microplate Reader. The experiment was repeated five times.

**Fig.S2. Confocal microscopy observations of endocytosed Dil-ac-LDL in the inhibitor-treated BmN cells**

BmN cells were treated with chlorpromazine (2 mM) (a), dansylcadaverine (2 mM) (b), or PP2 (0.16 μM) (d) for 30 min, or genistein (50 μg/mL) (c) for 1 h, followed by incubation with10 μg/mL Dil-ac-LDL (red); untreated cells were used as a control (e). Two hours after incubation, the cells were washed by PBS for 3 times. Then the cells were incubated with 5 μM Dio (Green) for 10 mins, images were captured by confocal microscopy. The nucleus was stained with DAPI (blue).

**Fig. S3. Effects of silencing the *AP*-1 and *clathrin* genes of BmN cells and silkworms.**

a, The relative expression level of the *AP*-1 in BmN cells at 48 h post-treatment with the *AP*-1-specific siRNAs AP-315, AP-1597, and AP-2014; b, The relative expression level of the *clathrin* gene in BmN cells at 48 h post- treatment with the clathrin-specific siRNAs clathrin-348, clathrin-517, and clathrin-1405; c, The relative expression level of the *AP*-1 gene in the midgut of silkworm at 48 h post-injection of the AP-315 siRNA; d, The relative expression level of the *clathrin* gene in the midgut of silkworm at 48 h post-injection of the clathrin-348 siRNA. Error bars indicate standard deviations. *, P < 0.5; **, p<0.01; ***, P< 0.001. e, The level of the clathrin and AP-1 proteins in BmN cells at 48 h post-treatment with the clathrin-348 siRNA and the AP-315 siRNA, respectively. Western blotting was performed using a mouse anti-clathrin or anti-AP-1 antibody as the primary antibody and a horseradish peroxidase-conjugated goat anti-mouse IgG (Tiangen, Beijing, China) as the secondary antibody. si-GFP, GFP-specific siRNA; si-CLAT, clathrin-specific siRNA clathrin-348; si-AP-1, *AP*-1-specific siRNA AP-315. The images were collected by ChemiScope6300. The blot of CLAT with Tublin or AP-1 with Tublin was cropped from different parts of the same gel with the optional exposure and the blot of Tublin as the internal reference to evaluate the amount of the total protein.

**Fig. S4. Entry of AP-1 and clathrin-specific antibody into BmN cells.**

a, BmN cells were treated with normal mouse serum at final concentration of 1000 μg/mL for 1h; b, BmN cells were treated with anti-AP-1 antibody at final concentration of 1000 μg/mL for 1h; c, BmN cells were treated with anti-clathrin antibody at final concentration of 1000 μg/mL for 1h. The treated cells were washed with PBS and fixed with 4 % paraformaldehyde for 5 min. Subsequently, the immunofluorescent staining was followed and observed under a confocal microscope. The anti-AP-1 or anti-clathrin antibodies were used as the primary antibodies, FITC labeled goat anti-mouse IgG (H+L) was used as the second antibody. The nucleus was stained with DAPI.

**Fig.S5. Confocal microscopy observations of endocytosed Dil-ac-LDL in the antibody-treated BmN cells**

a, BmN cells were treated with normal mouse serum at final concentration of 300 μg/mL for 1h; b, BmN cells were treated with anti-AP-1 antibody at final concentration of 300 μg/mL for 1h; c, BmN cells were treated with anti-clathrin antibody at final concentration of 300 μg/mL for 1h. The treated cells were followed by incubation with10 μg/mL Dil-ac-LDL (red) for 2 h. Then the cells were washed by PBS for 3 times and were incubated with 5 μM Dio (green) for 10 mins, images were captured by a confocal microscopy. The nucleus was stained with DAPI (blue).

**Fig. S6. Confocal microscopy observations of endocytosed BmCPV particles.**

a, BmN cells (control) were incubated with A546-labeled BmCPV virions for 3 h; b, BmN cells were treated with PP2 (0.16 μM) for 30 min, followed by incubation with A546-labeled BmCPV virions for 3 h; c, BmN cells were treated with genistein (25 μg/mL) for 1 h, followed by incubation with A546-labeled BmCPV virions for 3 h; d, BmN cells were treated with leupeptin hemisulfate (50 μM) for 30 min, followed by incubation with A546-labeled BmCPV virions for 3 h; e, BmN cells were treated initially with leupeptin hemisulfate (50 μM) for 30 min, and then with PP2 (0.16 μM) for 30 min, followed by incubation with A546-labeled BmCPV virions for 3 h; f, BmN cells were treated initially with leupeptin hemisulfate (50 μM) for 30 min, and then with genistein (50 μg/mL) for 1 h, followed by incubation with A546-labeled BmCPV virions for 3 h; g, Quantification of BmCPV particles in single planes of view (n = eight cells). Error bars indicate standard deviations. *, P < 0.5; **, P < 0.01; ***, P < 0.001.

Figure S1


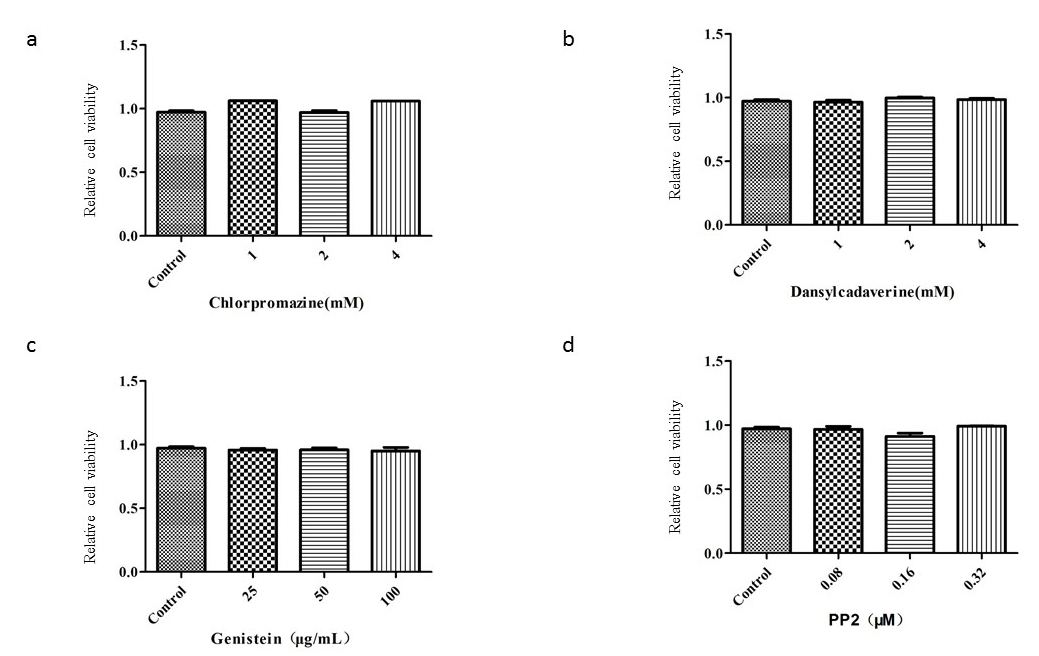


Figure S2


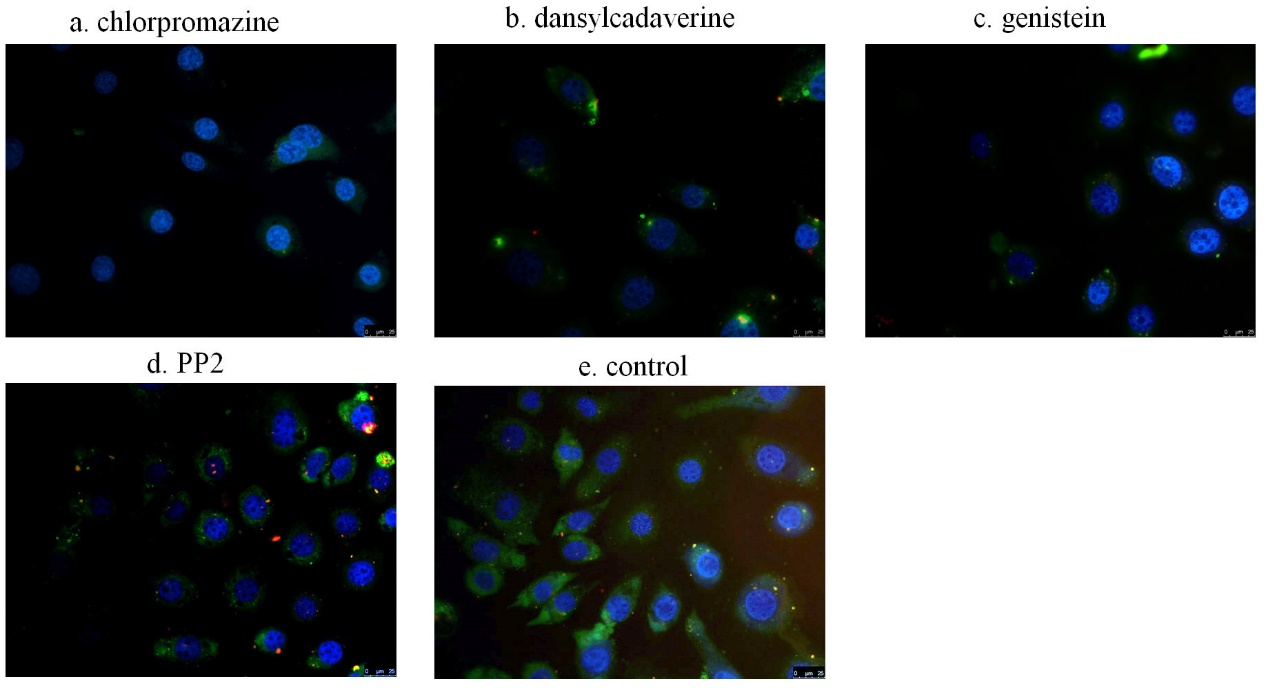


Figure S3


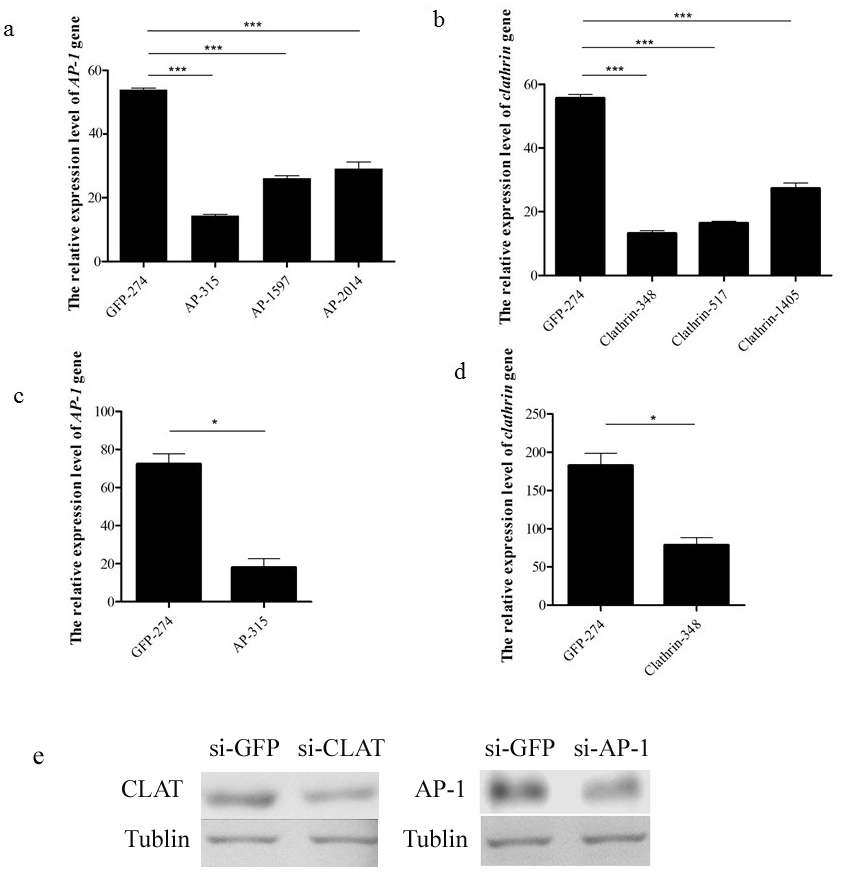


Figure S4


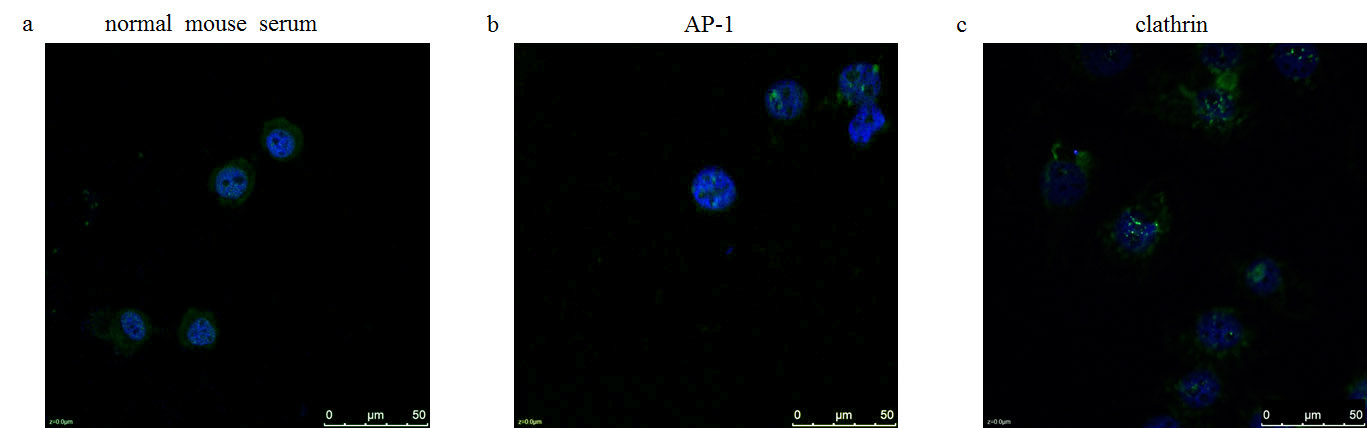


Figure S5


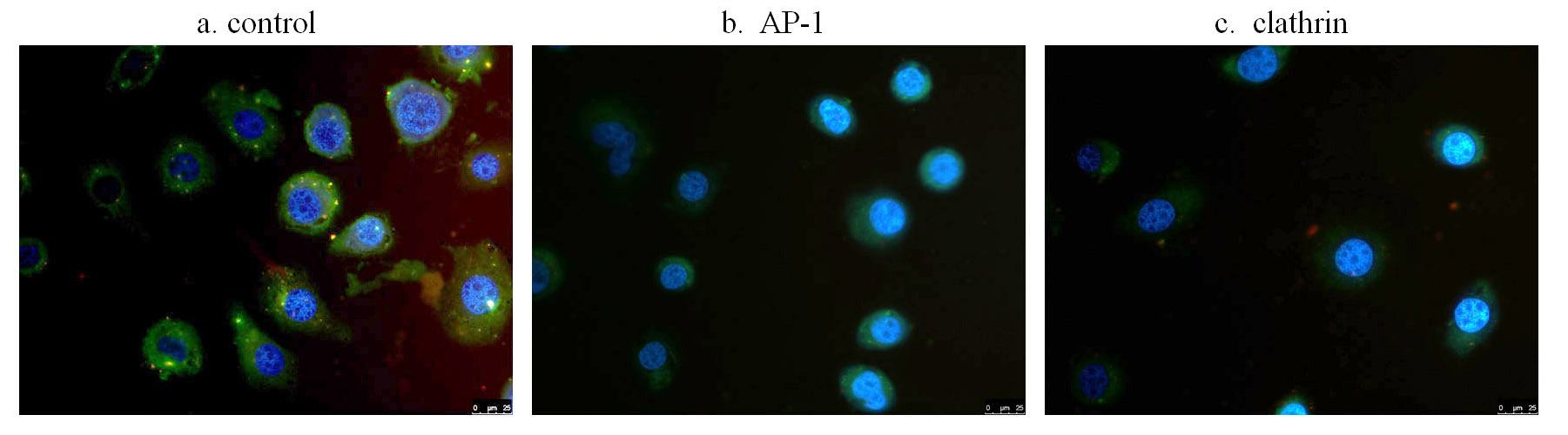


Figure S6


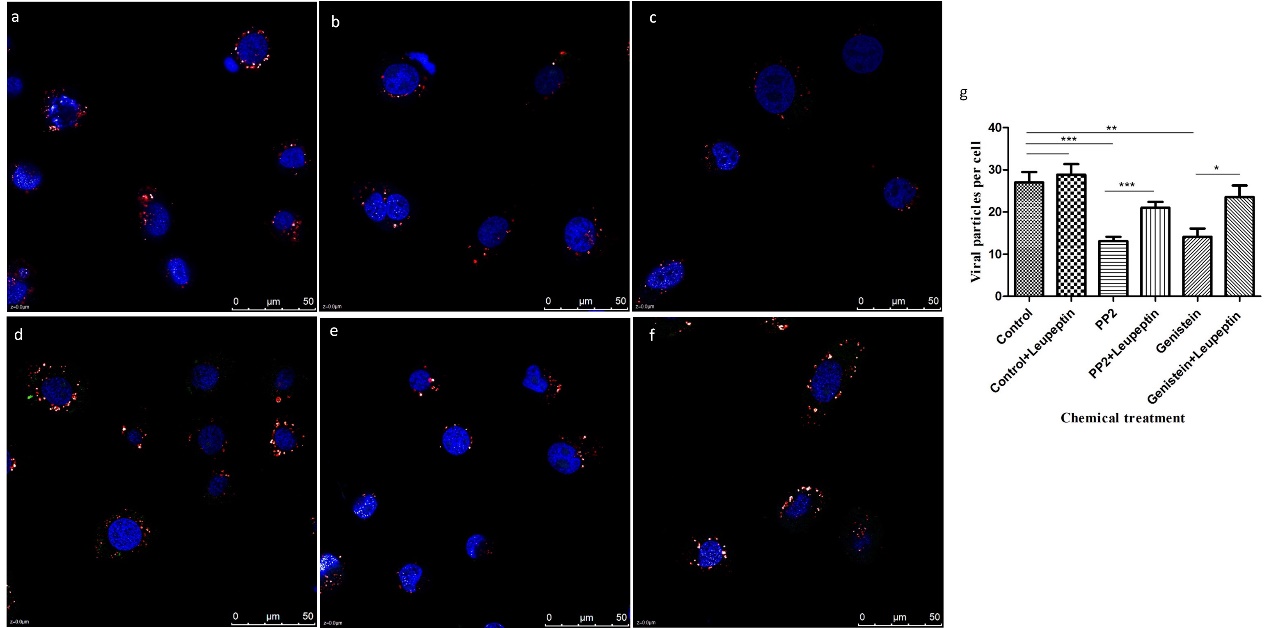


Table S1. The sequence of the siRNAs.

| Gene | siRNA | Sequence | |
| --- | --- | --- | --- |
|  |  | Positive-sense（5'-3'） | antisense（5'-3'） |
| *AP* | AP-315 | GCUUAAAGAACGACCUCAACA | UUGAGGUCGUUCUUUAAGCAG |
|  | AP-1597 | GCUCUCGAUCAGCACUAAAGA | UUUAGUUGCUGAUCGAGAGCUG |
|  | AP-2014 | GCUGAGCGGUCUAGAUCUAAC | UAGAUCUAGACCGCUCAGCAA |
| *clathrin* | Clathrin-348 | GGAAGUGGAUUUCACUGAACA | UUCAGUGAAAUCCACUUCCAG |
|  | Clathrin-517 | GGUCGGUAUUUCUGCUCAACA | UUGAGCAGAAAUACCGACCAG |
|  | Clathrin-1405 | CGGAGAAUCUUGUAAAGCAAGU | UUGCUUUACAAGAUCUCCGAG |
| *GFP* | GFP-274 | GGCUACGUCCAGGAGCGCACC | UGCGCUCCUGGACGUAGCCUU |

Table S2. Primers for qRT- PCR.

|  | Primer Sequence | |
| --- | --- | --- |
| Target genes | Forward primer（5'-3'） | Reverse primer（5'-3'） |
| *Actin A*3 | AACACCCCGTCCTGCTCACTG | GGGCGAGACGTGTGATTTCCT |
| *AP-1* | GCGTCGTCAACAAGGAATGCG | CAGTTCGTGATGAGGAGATGG |
| *clathrin* | GCGAGAAGGTTGGTGAGACTG | GTGACCAGAGCGAGTGTGTTC |
| *BmCPV vp1* | GGTCTCGACGTGAATACCGA | TCGTCTGCTTCACTAGCACG |

Table S3. Primers for prokaryotic expression.

| Target genes | Primer sequences | |
| --- | --- | --- |
|  | forward primer（5'-3'） | reverse primer（5'-3'） |
| *clathrin* | GGATCCCTGGTCGGTATTTCTGCTCAAC (*Bam*HI) | AAGCTTGCTGGATAGTTTGCGGTGTAC (*Hin*d III) |
| *AP* | GGATCCAGCGTCGTCAACAAGGAATGC (*Bam*HI) | AAGCTTGCCGCCTTCTTCTTTATGTAG (*Hin*dIII) |
